# Long-Term Survival in Patients With Advanced Melanoma

**DOI:** 10.1001/jamanetworkopen.2024.26641

**Published:** 2024-08-14

**Authors:** Olivier J. van Not, Alfons J. M. van den Eertwegh, Hilde Jalving, Manja Bloem, John B. Haanen, Rozemarijn S. van Rijn, Maureen J. B. Aarts, Franchette W. P. J. van den Berkmortel, Christian U. Blank, Marye J. Boers-Sonderen, Jan Willem de Groot J. W. B., Geke A. P. Hospers, Ellen Kapiteijn, Brenda Leeneman, Piersma D., Marion Stevense-den Boer, Astrid A. M. van der Veldt, Gerard Vreugdenhil G., Michel W. J. M. Wouters, Willeke A. M. Blokx, Karijn P. M. Suijkerbuijk

**Affiliations:** 1 Scientific Bureau, Dutch Institute for Clinical Auditing, Leiden, the Netherlands; 2 Department of Medical Oncology, University Medical Center Utrecht, Utrecht University, Utrecht, the Netherlands; 3 Department of Medical Oncology, Amsterdam UMC, VU University Medical Center, Cancer Center Amsterdam, Amsterdam, the Netherlands; 4 Department of Medical Oncology, University Medical Center Groningen, University of Groningen, Groningen, the Netherlands; 5 Department of Biomedical Data Sciences, Leiden University Medical Center, Leiden, the Netherlands; 6 Department of Surgical Oncology, Netherlands Cancer Institute, Amsterdam, the Netherlands; 7 Department of Molecular Oncology and Immunology, Netherlands Cancer Institute, Amsterdam, the Netherlands; 8 Department of Internal Medicine, Medical Center Leeuwarden, Leeuwarden, the Netherlands; 9 Department of Medical Oncology, GROW School for Oncology and Developmental Biology, Maastricht University Medical Center+, Maastricht, the Netherlands; 10 Department of Medical Oncology, Zuyderland Medical Center Sittard, Sittard-Geleen, the Netherlands; 11 Department of Medical Oncology and Immunology, Netherlands Cancer Institute, Amsterdam, the Netherlands; 12 Department of Medical Oncology, Radboud University Medical Center, Nijmegen, the Netherlands; 13 Isala Oncology Center, Isala, Zwolle, the Netherlands; 14 Department of Medical Oncology, Leiden University Medical Center, Leiden, the Netherlands; 15 Department of Health Technology Assessment, Erasmus School of Health Policy and Management, Erasmus University Rotterdam, Rotterdam, the Netherlands; 16 Erasmus Center for Health Economics Rotterdam, Erasmus University Rotterdam, Rotterdam, the Netherlands; 17 Department of Internal Medicine, Medisch Spectrum Twente, Enschede, the Netherlands; 18 Department of Internal Medicine, Amphia Hospital, Breda, the Netherlands; 19 Department of Medical Oncology and Radiology and Nuclear Medicine, Erasmus Medical Center, Rotterdam, the Netherlands; 20 Department of Internal Medicine, Maxima Medical Center, Eindhoven, the Netherlands; 21 Department of Pathology, University Medical Center Utrecht, Utrecht University, Utrecht, the Netherlands

## Abstract

**IMPORTANCE:**

Long-term survival data from clinical trials show that survival curves of patients with advanced melanoma treated with immune checkpoint inhibitors (ICIs) gradually reach a plateau, suggesting that patients have a chance of achieving long-term survival.

**OBJECTIVE:**

To investigate long-term survival in patients with advanced melanoma treated with ICIs outside clinical trials.

**DESIGN, SETTING, AND PARTICIPANTS:**

Cohort study using prospectively collected data from the nationwide Dutch Melanoma Treatment Registry, including patients in the Netherlands with advanced melanoma treated with first-line ICIs from 2012 to 2019. Data were analyzed from January to September 2023.

**EXPOSURES:**

Patients were treated with first-line ipilimumab-nivolumab, antibodies that target programmed cell death (anti–PD-1), or ipilimumab.

**MAIN OUTCOMES AND MEASURES:**

Progression-free survival (PFS) and melanoma-specific survival were analyzed, and a Cox proportional hazards model was used to investigate factors associated with PFS after reaching partial response (PR) or complete response (CR).

**RESULTS:**

A total of 2490 patients treated with first-line ICIs were included (median [IQR] age, 65.0 [55.3-73.0] years; 1561 male patients [62.7%]). Most patients had an Eastern Cooperative Oncology Group Performance Status of 1 or lower (2202 patients [88.5%]) and normal lactate dehydrogenase levels (1715 patients [68.9%]). PFS for all patients was 23.4% (95% CI, 21.7%-25.2%) after 3 years and 19.7% (95% CI, 18.0%-21.4%) after 5 years. Overall survival for all patients was 44.0% (95% CI, 42.1%-46.1%) after 3 years and 35.9% (95% CI, 33.9%-38.0%) after 5 years. Patients with metastases in 3 or more organ sites had a significantly higher hazard of progression after reaching PR or CR (adjusted hazard ratio, 1.37; 95% CI, 1.11-1.69).

**CONCLUSIONS AND RELEVANCE:**

This cohort study of patients with advanced melanoma treated with ICIs in clinical practice showed that their survival reached a plateau, comparable with patients participating in clinical trials. These findings can be used in daily clinical practice to guide long-term surveillance strategies and inform both physicians and patients regarding long-term treatment outcomes.

## Key Points

**Question:**

What are the long-term outcomes of patients with advanced melanoma who are treated in daily clinical practice, and do they have a chance of sustaining long-term survival?

**Findings:**

In this cohort study including 2490 patients with advanced melanoma treated with first-line immune checkpoint inhibitors, survival curves flattened over time, suggesting long-term survival.

**Meaning:**

These findings suggest patients treated in clinical practice for advanced melanoma may reach a plateau phase in their survival after treatment with immune checkpoint inhibitors, as is seen in clinical trials.

## Teaser

This cohort study evaluates long-term survival in patients with advanced melanoma treated with immune checkpoint inhibitors outside clinical trials.

## Introduction

Before the introduction of new therapeutic agents, such as immune checkpoint inhibitors (ICIs) and *BRAF/MEK* inhibitors, the median overall survival (OS) of patients with advanced melanoma was less than a year with only 1 in 10 patients surviving more than 5 years.^1^ The introduction of these new systemic treatments has significantly improved the prognosis of patients with advanced melanoma.^2^ Durable responses reported in clinical trials were also observed in patients treated in clinical practice.^3,4^ Ipilimumab, a monoclonal antibody blocking* CTLA-4*, was introduced in 2012 and was the first registered ICI for the treatment of advanced melanoma.^5^ Antibodies that target programmed cell death (anti–PD-1) were introduced in 2015, and the combination of ipilimumab and nivolumab in 2016.^6,7^ Targeted therapies, such as *BRAF* inhibitor therapies, were introduced in 2012, whereas *MEK* inhibitors were introduced in 2016 in the Netherlands.^8,9^

In 2015, Schadendorf et al^10^ published a pooled analysis of the long-term survival data from phase 2 and 3 trials investigating 1861 patients treated with ipilimumab in advanced melanoma. Instead of the survival curves declining until no patients were left, they observed a plateau in the survival curves. Updated long-term survival data on phase 1 through 3 studies investigating anti–PD-1 and ipilimumab-nivolumab were recently published.^11,12,13,14,15,16^ These studies investigating patients with advanced melanoma reported on long-term survival outcomes and showed that survival curves also eventually reached a plateau, meaning that patients who respond to ICIs have a chance of sustaining long-term OS. Data on long-term outcomes of patients treated with ICIs in the clinical practice setting are limited. Prior studies have shown that patients who receive treatment in trials have better disease characteristics and survival outcomes than those treated in clinical practice.^17,18^ Therefore, it is important to determine whether patents treated with ICIs in the clinical practice setting can also achieve long-term survival.

Based on data from clinical trials, the question was raised whether these durable responses may actually be cures.^19^ In the Netherlands, patients who remain progression-free for 5 years after reaching a confirmed partial or complete response have recently been advised to stop standard radiological follow-up.^20^

The aims of this study were to investigate the long-term survival outcomes of patients treated with ICIs in clinical practice, to evaluate whether the survival curves show a plateau phase, and to evaluate progression-free survival (PFS) after reaching partial or complete response to ICIs. Moreover, we aimed to identify factors associated with progression after initial response.

## Methods

For this cohort study, we retrieved data from the Dutch Melanoma Treatment Registry (DMTR). The DMTR registers data collected for all systemically treated patients with stage 3 and 4 melanoma in the Netherlands, and data collection commenced in 2012.^21^ We included all patients with advanced (ie, irresectable or metastatic) cutaneous melanoma who were treated with first-line ICIs (ipilimumab monotherapy, anti–PD-1, or ipilimumab-nivolumab). Patients with uveal or mucosal melanoma and patients with missing values required for the survival analysis were excluded. We included patients starting ICI treatment between 2012 and 2019 to ensure that patients had a minimum of 3 years of follow-up. We analyzed the best objective response rate (BORR), PFS, OS, and melanoma-specific survival (MSS). We further stratified and investigated patients by type of treatment: ipilimumab, anti–PD-1, and ipilimumab-nivolumab. The treating physician determined the response evaluation in line with the Response Evaluation Criteria in Solid Tumors version 1.1.^22^

The medical ethical committee of Leiden University Medical Center approved research using DMTR data and concluded that this research was not subject to the Medical Research Involving Human Subjects Act in compliance with Dutch regulations, so informed consent was not required. For this study, the dataset cutoff date was July 3, 2023. This study followed Strengthening the Reporting of Observational Studies in Epidemiology (STROBE) reporting guidelines.

### Patient Characteristics, Tumor Characteristics, and Treatment Outcomes

Patient and tumor characteristics collected and analyzed for all patients at baseline were: age at diagnosis, sex, Eastern Cooperative Oncology Group Performance Status (ECOG PS), lactate dehydrogenase levels (LDH), primary melanoma location, liver metastasis, brain metastasis, number of organ sites with metastases, stage according to American Joint Committee on Cancer (AJCC) 8th edition,^23^ and *BRAF* and *NRAS* variant status.

### Statistical Analysis

We used descriptive statistics to analyze baseline characteristics. Categorical variables were compared using the Pearson χ^2^ test, and continuous variables were compared using the *t* test or Mann-Whitney U test depending on whether the variables were normally distributed. The baseline characteristics were compared across the different treatment groups. Median follow-up was estimated using the reversed Kaplan-Meier method.^24^ We calculated the BORR for the first systemic treatment line. The BORR was defined as the proportion of patients who achieved a complete response (CR) or partial response (PR). The Kaplan-Meier method was used to calculate the median PFS and OS. PFS was defined as start of systemic therapy for advanced melanoma to first progression or death. OS was defined as the start of systemic treatment to death by any cause. MSS was defined as the start of systemic treatment to melanoma-related death. Patients not experiencing an event were right-censored at the last contact date. We performed additional analyses for patients developing a PR or CR as best response during their first-line treatment with ICIs. To allow comparison with data reported from relevant clinical trials,^25^ we calculated PFS and OS starting from the date of partial or complete response. Moreover, we used a Cox proportional hazards model to perform a multivariable regression analysis to assess patient and tumor-related factors associated with PFS after PR or CR. Covariates used were age, sex, ECOG PS, LDH levels, brain metastases, liver metastases, number of organ sites with metastases, year of ICI treatment, *BRAF* variant status, and type of systemic treatment. Comparisons were considered statistically significant for 2-sided *P* values less than .05. Analysis were completed using R studio version 4.0.2 (R Project for Statistical Computing)^26^ with the tableone,^27^ survival,^28^ and survminer^29^ packages. Data were analyzed from January to September 2023.

## Results

Between 2012 and 2019, 2652 patients with advanced melanoma treated with first-line ICIs were registered in the DMTR database (median [IQR] age, 65.0 [55.3-73.0] years; 1561 male patients [62.7%]). We excluded 2 patients with missing values needed for survival analyses, 58 patients with uveal melanoma, and 102 patients with mucosal melanoma. Of the 2490 included patients, 601 were treated with ipilimumab monotherapy, 1409 with anti–PD-1, and 480 with ipilimumab-nivolumab. Median follow-up for the entire cohort was 55.8 (95% CI, 53.6-57.5) months. Median follow-up was 89.1 (95% CI, 86.7-93.7) months for patients treated with ipilimumab monotherapy, 52.9 (95% CI, 51.2-55.0) months for patients treated with anti–PD-1, and 46.5 (95% CI, 44.5-48.5) months for patients treated with ipilimumab-nivolumab.

### Patient Characteristics

A total of 2202 patients (88.5%) had an ECOG PS of 1 or lower and normal LDH levels (1715 patients [68.9%]). Liver metastases were present in 626 patients (25.1%), and brain metastases in 565 (22.7%). *BRAF* variants were present in 980 patients (39.4%), and *NRAS* variants in 719 (28.9%). Most patients were treated with anti–PD-1 monotherapy (1409 patients [56.6%]), followed by ipilimumab (601 patients [24.1%]), and ipilimumab-nivolumab (480 patients [19.3%]). In general, patients treated with ipilimumab-nivolumab had poorer disease characteristics than patients treated with ipilimumab or anti–PD-1. All patient and tumor characteristics can be found in the Table. A total of 606 patients (24.3%) reached PR, and 504 patients (20.2%) reached CR. BORR for the entire cohort was 44.5%. BORR was 17.5% following ipilimumab monotherapy, 53.1% following anti–PD-1, and 53.3% following ipilimumab-nivolumab (eTable 1 in Supplement 1).

**Table.** Patient, Tumor, and Treatment Characteristics of Patients With Advanced Melanoma Treated With Immune Checkpoint Inhibitors (ICIs)

Characteristic	Patients, No. (%)			
	Ipilimumab (n = 601)	Anti–PD-1 (n = 1409)	Ipilimumab-nivolumab (n = 480)	Total (N = 2490)
Age, y				
<65	325 (54.1)	556 (39.5)	299 (62.3)	1180 (47.4)
≥65	276 (45.9)	853 (60.5)	181 (37.7)	1310 (52.6)
Age, median (IQR), y	63.0 (53.0-70.0)	68.0 (58.0-76.0)	61.0 (51.0-70.0)	65.0 (55.3-73.0)
Sex				
Male	383 (63.7)	869 (61.7)	309 (64.4)	1561 (62.7)
Female	218 (36.3)	540 (38.3)	171 (35.6)	929 (37.3)
ECOG PS				
0	383 (63.7)	789 (56.0)	229 (47.7)	1401 (56.3)
1	158 (26.3)	451 (32.0)	192 (40.0)	801 (32.2)
≥2	15 (2.5)	85 (6.0)	35 (7.3)	135 (5.4)
Unknown	45 (7.5)	84 (6.0)	24 (5.0)	153 (6.1)
LDH levels				
Not determined	17 (2.8)	20 (1.5)	6 (1.2)	43 (1.7)
Normal	455 (75.7)	1042 (74.0)	218 (45.4)	1715 (68.9)
1-2x ULN	103 (17.1)	294 (20.9)	155 (32.3)	552 (22.2)
>2x ULN	26 (4.3)	53 (3.8)	101 (21.0)	180 (7.2)
Melanoma location				
Primary unknown	99 (16.5)	201 (14.3)	83 (17.3)	383 (15.4)
Head-neck	76 (12.6)	234 (16.6)	70 (14.6)	380 (15.3)
Trunk	203 (33.8)	485 (34.4)	186 (38.8)	874 (35.1)
Extremities	189 (31.4)	431 (30.6)	127 (26.5)	747 (30.0)
Acral	29 (4.8)	51 (3.6)	8 (1.7)	88 (3.5)
Unknown	5 (0.8)	7 (0.5)	6 (1.2)	18 (0.7)
Liver metastases				
No	445 (74.0)	1103 (78.3)	291 (60.6)	1839 (73.9)
Yes	146 (24.3)	294 (20.9)	186 (38.8)	626 (25.1)
Unknown	10 (1.7)	12 (0.9)	3 (0.6)	25 (1.0)
Brain metastases				
No	481 (80.0)	1162 (82.5)	274 (57.1)	1917 (77.0)
Yes, asymptomatic	67 (11.1)	146 (10.4)	118 (24.6)	331 (13.3)
Yes, symptomatic	49 (8.2)	100 (7.1)	85 (17.7)	234 (9.4)
Unknown	4 (0.7)	1 (0.1)	3 (0.6)	8 (0.3)
AJCC stage (8th edition)				
III irresectable	19 (3.2)	119 (8.4)	17 (3.5)	155 (6.2)
IV-M1a	76 (12.6)	165 (11.7)	16 (3.3)	257 (10.3)
IV-M1b	99 (16.5)	251 (17.8)	26 (5.4)	376 (15.1)
IV-M1c	287 (47.8)	627 (44.5)	215 (44.8)	1129 (45.3)
IV-M1d	116 (19.3)	246 (17.5)	203 (42.3)	565 (22.7)
Unknown	4 (0.7)	1 (0.1)	3 (0.6)	8 (0.3)
Organ sites				
<3	337 (56.1)	861 (61.1)	193 (40.2)	1391 (55.9)
≥3	262 (43.6)	542 (38.5)	284 (59.2)	1088 (43.7)
Unknown	2 (0.3)	6 (0.4)	3 (0.6)	11 (0.4)
Variant				
* BRAF*	224 (37.3)	556 (39.5)	200 (41.7)	980 (39.4)
* NRAS*	152 (25.3)	421 (29.9)	146 (30.4)	719 (28.9)
Targeted therapy after ICI treatment				
No	455 (75.7)	1105 (78.4)	389 (81.0)	1949 (78.3)
Yes	146 (24.3)	304 (21.6)	91 (19.0)	541 (21.7)
ICIs after ICI treatment				
No	347 (57.7)	1023 (72.6)	398 (82.9)	1768 (71.0)
Yes	254 (42.3)	386 (27.4)	82 (17.1)	722 (29.0)
Maximum No. of systemic treatment lines				
1	219 (36.4)	837 (59.4)	315 (65.6)	1371 (55.1)
2	202 (33.6)	322 (22.9)	110 (22.9)	634 (25.5)
3	106 (17.6)	134 (9.5)	36 (7.5)	276 (11.1)
4	49 (8.2)	73 (5.2)	10 (2.1)	132 (5.3)
≥5	25 (4.2)	43 (3.1)	9 (1.9)	77 (3.1)

Abbreviations: AJCC, American Joint Committee on Cancer; EGOG PS, Eastern Cooperative Oncology Group Performance Status; LDH, lactate dehydrogenase; ULN, upper limits of normal.

### Survival Outcomes

Median PFS for all patients was 5.7 (95% CI, 5.5-6.3) months. The 2-year, 3-year, and 5-year PFS rates were 27.1% (95% CI, 25.4%-29.0%), 23.4% (95% CI, 21.7%-25.2%), and 19.7% (95% CI, 18.0%-21.4%), respectively. Median OS was 25.1 (95% CI, 22.6-28.8) months. The 2-year OS rate was 50.9% (95% CI, 48.9%-52.9%), the 3-year OS rate was 44.0% (95% CI, 42.1%-46.1%), and the 5-year OS rate was 35.9% (95% CI, 33.9%-38.0%). Median MSS was 29.9 (95% CI, 26.7-34.2) months with a 5-year MSS rate of 39.2% (95% CI, 37.1%-41.4%).

**Figure 1.**
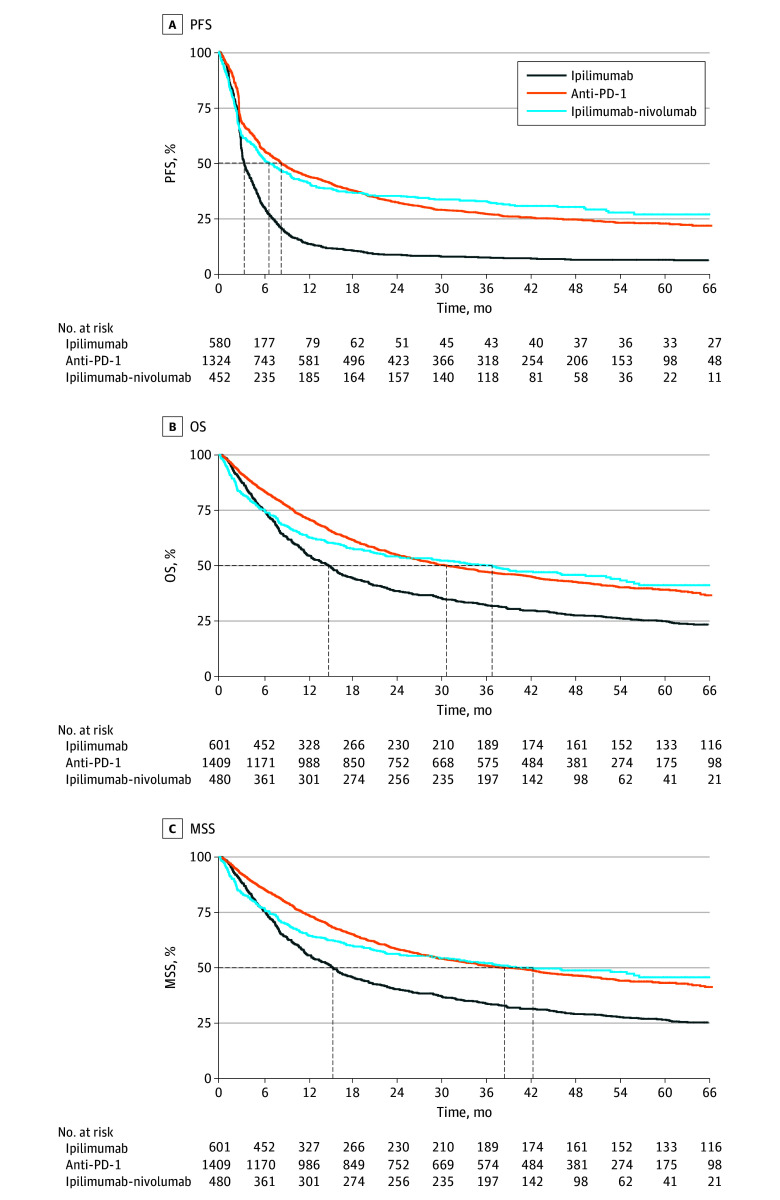
Kaplan-Meier Curves of Progression-Free Survival (PFS), Overall Survival (OS), and Melanoma-Specific Survival (MSS) Anti–PD-1 indicates antibodies that target programmed cell death.

Median PFS was lowest for the ipilimumab cohort (median PFS, 3.4; 95% CI, 3.2-3.9 months). Median PFS was 8.4 (95% CI, 7.3-9.8) months for the anti–PD-1 cohort and 6.8 (95% CI, 5.4-9.4) months for the ipilimumab-nivolumab cohort (Figure 1A). The 5-year PFS rate for patients treated with ipilimumab was 6.5% (95% CI, 4.8%-8.9%), the rate for patients treated with anti–PD-1 was 22.8% (95% CI, 20.5%-25.3%), and the rate for patients treated with ipilimumab-nivolumab was 26.9% (95% CI, 22.5%-32.2%).

The same results were seen for OS, with a median OS of 14.8 (95% CI, 12.5-16.7) months for patients treated with ipilimumab, 30.7 (95% CI, 27.0-35.8) months for patients treated with anti–PD-1, and 36.8 (95% CI, 22.6-52.1) months for patients treated with ipilimumab-nivolumab (Figure 1B). The 5-year OS rate for patients treated with ipilimumab was 24.9% (95% CI, 21.7%-28.7%), the rate for patients treated with anti–PD-1 was 39.1% (95% CI, 36.4%-42.1%), and the rate for patients treated with ipilimumab-nivolumab was 41.2% (95% CI, 3.1%-44.9%). Median MSS was 15.4 (95% CI, 13.0-18.0) months for the ipilimumab cohort, 38.4 (95% CI, 33.0-45.6) months for the anti–PD-1 cohort, and 42.3 (95% CI, 29.8-not reached [NR]) months for the ipilimumab-nivolumab cohort (Figure 1C). The plateau phase in the survival curves visually appears more pronounced for patients treated with ipilimumab-nivolumab than for patients treated with anti–PD-1.

### Survival Outcomes for Patients With a Partial or Complete Response

Baseline characteristics of patients reaching complete or partial response vs those who did not reach complete or partial response are presented in eTable 2 in Supplement 1. Patients reaching complete or partial response had significantly lower LDH levels, had lower ECOG scores, were less likely to have liver or brain metastases, were less likely to have more than 3 organ sites with metastases, and thus had a lower disease stage. In the cohort of patients with PR, the median PFS was 13.8 (95% CI, 11.6-15.9) months calculated from the first date of PR. Median PFS from date of first PR was 4.5 (95% CI, 3.2-6.2) months for the ipilimumab cohort, 14.7 (95% CI, 12.9-16.8) months for the anti–PD-1 cohort, and 22.0 (95% CI, 10.9-33.9) months for the ipilimumab-nivolumab cohort. PFS-landmark analyses from first PR showed a 2-year PFS of 35.5% (95% CI, 31.7%-39.8%), and a 3-year PFS of 27.3% (95% CI, 23.8%-31.5%). Median OS from PR was 45.6 (95% CI, 40.2-53.8) months. OS-landmark analyses from first PR showed a 2-year OS of 67.2% (95% CI, 63.5%-71.1%) and a 3-year OS of 57.2% (95% CI, 53.2%-61.4%).

In the cohort of patients with CR, median PFS was 65.2 (95% CI, 56.6-83.2) months calculated from the first date of CR. Median PFS from the date of the first CR was 83.2 (95% CI, 61.9-NR) months for the ipilimumab cohort, 65.2 (95% CI, 55.2-NR) months for the anti–PD-1 cohort, and 55.0 (95% CI, 44.0-NR) months for the ipilimumab-nivolumab cohort. PFS-landmark analyses from first CR showed a 2-year PFS of 79.7% (95% CI, 75.9%-83.6%) and a 3-year PFS of 72.4% (95% CI, 68.0%-77.0%). Two-year PFS was 83.5% (95% CI, 73.2%-96.5%) for ipilimumab, 77.3% (95% CI, 72.8%-82.1%) for anti–PD-1, and 87.3% (95% CI, 80.0%-95.1%) for ipilimumab-nivolumab. OS-landmark analyses from first CR showed a 2-year OS of 94.0% (95% CI, 91.9%-96.3%) and a 3-year OS of 90.3% (95% CI, 87.4%-93.3%).

PFS and OS calculated from date of best response stratified by treatment type (anti–PD-1 or ipilimumab-nivolumab) and best response (CR, PR, or stable disease) can be found in eFigure 1, eFigure 2, eFigure 3, and eFigure 4 in Supplement 1. Median MSS was 75.9 (95% CI, 58.3-NR) months for patients with PR and was not reached for patients with CR.

**Figure 2.**
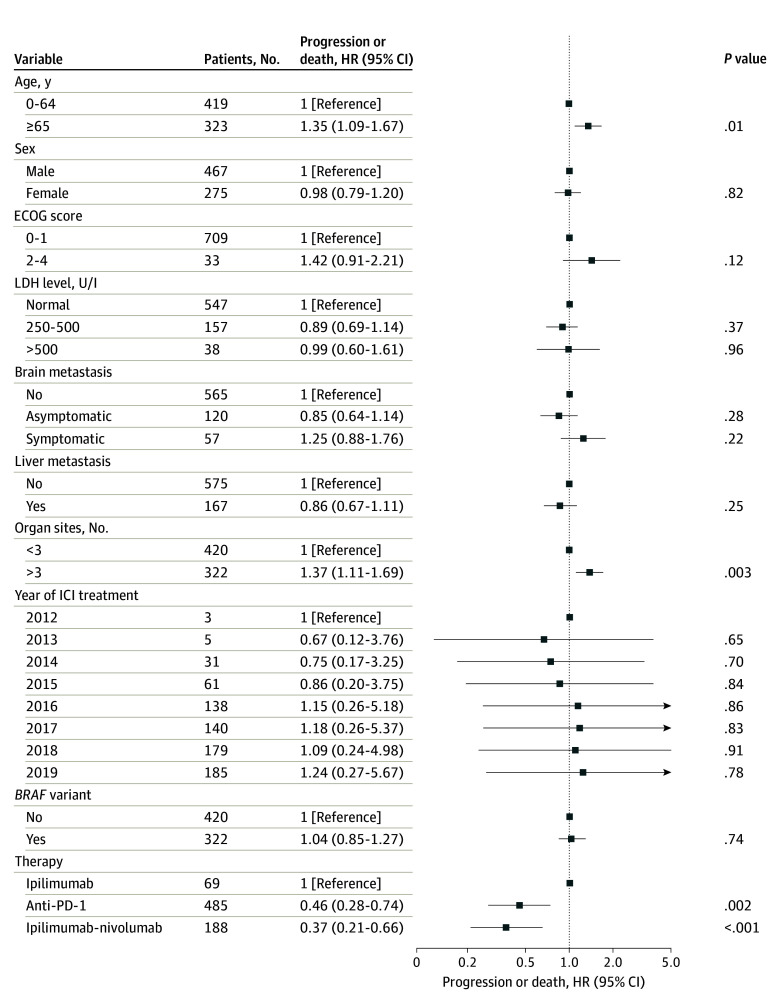
Multivariable Cox Regression Model of Factors Associated With Progression or Death After Reaching Partial or Complete Response Anti–PD-1 indicates antibodies that target programmed cell death; ECOG, Eastern Cooperative Oncology Group; HR, hazard ratio; ICI, immune checkpoint inhibitor; LDH, lactate dehydrogenase.

In patients reaching PR or CR, being aged 65 years or older (adjusted hazard ratio [aHR], 1.35; 95% CI, 1.09-1.67) and having more than 3 organ sites with metastases (aHR, 1.37; 95% CI, 1.11-1.69) were significantly associated with a higher adjusted hazard of progression or death after reaching PR or CR (Figure 2). Treatment with anti–PD-1 (aHR, 0.46; 95% CI, 0.28-0.74) or ipilimumab-nivolumab (aHR, 0.37; 95% CI, 0.21-0.66) was associated with a lower odds of progression compared with ipilimumab in patients reaching PR or CR.

## Discussion

The present study is, to our knowledge, the largest population-based study to investigate long-term survival outcomes in patients with advanced melanoma treated with first-line ICIs. More than 70% of the patients developing a CR during their first-line treatment with ICIs had a PFS of more than 3 years, and more than 90% survived more than 3 years calculated from the date of CR. Interestingly, although no formal statistics were performed, the curve of the ipilimumab-nivolumab cohort appears to flatten more than the curves of the anti–PD-1 cohort. Based on the data, we cannot conclude if this points to a more durable response to ipilimumab-nivolumab compared with anti–PD-1 monotherapy or is explained by selection or indication bias in this clinical practice cohort.

As we described previously,^30^ survival outcomes in the first 2 years after start of treatment are inferior for the patients treated with ipilimumab-nivolumab compared with those treated with anti–PD-1 in our registry, which likely is explained by the worse disease characteristics of the ipilimumab-nivolumab cohort. We now see that these curves cross during the first years, suggesting that the plateau in patients treated in clinical practice is more prominent in patients receiving ipilimumab-nivolumab. Remarkably, the differences in the survival curves of anti–PD-1 and ipilimumab-nivolumab are less clear when evaluating MSS. This can indicate that competing causes of death, explained by the difference of 6 years in median age between the anti–PD-1 and ipilimumab-nivolumab cohorts, may also play a role in the differences in OS curves.

One recently published study^31^ has investigated the long-term survival outcomes of patients with advanced melanoma treated with ICIs in clinical practice, outside the setting of a clinical trial. This study included fewer patients than our study; 147 patients were treated with anti–PD-1, and 81 patients were treated with ipilimumab-nivolumab. They also reported superior long-term outcomes for patients treated with ipilimumab-nivolumab. Prior research using DMTR data has investigated the outcomes of patients with advanced melanoma treated with ipilimumab-nivolumab.^32^ They found higher survival rates for patients with PR and stable disease. However, they performed landmark analyses starting at 3 and 6 months, whereas our survival curves started at the time of best response. Performing the analysis of van Zeijl et al^32^ on our data yields comparable results (eFigure 5 and eFigure 6 in Supplement 1). Most data on long-term survival have been reported from clinical trials. Thus far, there is no prospective study that we know of that has shown a significant OS benefit of anti–PD-1 monotherapy over ipilimumab-nivolumab.

Schadendorf et al^10^ published a pooled analysis of phase 2 and 3 trials investigating ipilimumab in patients with advanced melanoma. Their analysis included 254 patients with at least 3 years of follow-up and showed a 26% 3-year survival rate for treatment-naive patients. An update on the CheckMate 067 trial^11,14^ reported the 5-year survival outcomes of treatment-naive patients treated with nivolumab, ipilimumab-nivolumab, or ipilimumab. They found better survival outcomes in the ipilimumab-nivolumab group than in the nivolumab and ipilimumab groups. Although not powered for a formal comparison between the 2 groups, this study suggested better survival outcomes in the ipilimumab-nivolumab group than in the nivolumab and ipilimumab groups. They reported a 5-year PFS of 36% in the ipilimumab-nivolumab group, 29% in the anti–PD-1 group, and 8% in the ipilimumab group. Five-year OS was 52% in the ipilimumab-nivolumab group, 44% in the nivolumab group and 26% in the ipilimumab group. These 5-year PFS and OS rates are higher than the 5-year rates we found, but patients who receive treatment in trials are known to have more favorable characteristics than those treated in clinical practice.^17^ Follow-up data from the CheckMate 066 trial^13^ reported on 5-year outcomes of patients with *BRAF* wild-type treated with nivolumab. Five-year OS rates were 39%, comparable with the 5-year OS we found in anti–PD-1-treated patients. Long-term outcomes of the KEYNOTE-001 trial published by Robert et al^25^ showed a 2-year disease-free survival rate from date of CR of 90.9% for patients treated with pembrolizumab. This is higher than the percentage (77.3%) we found in patients reaching CR. Of note, the percentage of patients reaching CR or PR in our cohort are higher than in clinical trials. This might indicate that indication bias or differences in response determination between trials and the clinical practice setting influence these outcomes. Comparison of PFS and OS rates in our study and in CheckMate 066, CheckMate 067, and KEYNOTE-001 can be found in eTable 3 in Supplement 1.

### Limitations

This study has some limitations. Compared with clinical trials, population-based studies are more prone to missing data. However, regularly trained, independent data managers register DMTR data, which are checked by the treating physicians to warrant the quality. Moreover, the online registry in which patients are registered warns data managers of inconsistent or missing values. The high quality of the data and the low number of missing values in the DMTR have been demonstrated by Jochems et al.^21^ Bias by indication may also play a role in observational studies such as the present one. The treating physician determines the given treatment together with the patient based on clinical characteristics. The observational nature of this study limits the conclusions that can be drawn in the comparison of the different treatments. Furthermore, to fully be able to evaluate patients reaching a plateau, a follow-up of at least 10 years is needed. Since the DMTR is the largest melanoma registry with nationwide inclusion of all patients with advanced melanoma, this registry is optimal for further studies investigating long-term survival of patients treated in clinical practice. We will continue to extend the follow-up of the patients included in this study to further evaluate their survival curves.

## Conclusions

The findings of our study are important for daily clinical practice and can be used to inform physicians and patients of the long-term treatment outcomes following ICI treatment in clinical practice settings. Moreover, these data can be used to develop follow-up plans for long-term survivors of advanced melanoma.

## Notes

Supplement 1. **eFigure 1. **Kaplan Meier Curve of Progression-Free Survival (PFS) of Patients With Advanced Melanoma Treated With Anti–PD-1, Stratified by Response, Calculated From the Date of Response **eFigure 2. **Kaplan Meier Curve of Overall Survival (OS) of Patients With Advanced Melanoma Treated With Anti–PD-1, Stratified by Response, Calculated From the Date of Response **eFigure 3. **Kaplan Meier Curve of Progression-Free Survival (PFS) of Patients With Advanced Melanoma Treated With Ipilimumab-Nivolumab, Stratified by Response, Calculated From the Date of Response **eFigure 4. **Kaplan Meier Curve of Overall Survival (OS) of Patients With Advanced Melanoma Treated With Ipilimumab-Nivolumab, Stratified by Response, Calculated From the Date of Response **eFigure 5. **Kaplan Meier Curve of Overall Survival (OS) of Patients With Advanced Melanoma Treated With Immune Checkpoint Inhibitors at Landmark Time From 3 Months After Start of Systemic Treatment, Stratified by Response Status at Landmark Time **eFigure 6. **Kaplan Meier Curve of Overall Survival (OS) of Patients With Advanced Melanoma Treated With Immune Checkpoint Inhibitors at Landmark Time From 6 Months After Start of Systemic Treatment, Stratified by Response Status at Landmark Time **eTable 1. **Best Objective Response Rate Stratified by Type of Systemic Treatment **eTable 2. **Patient and Tumor Characteristics of Patients With Advanced Melanoma Treated With Immune Checkpoint Inhibitors Stratified by Best Objective Response Rate: Complete or Partial Response vs No Complete or Partial Response **eTable 3. **Comparison of Findings of the Present Study With Other Relevant Trials
eReferences.

Supplement 2.
Data Sharing Statement
