# Supplementary material for: Long-Term Survival in Patients With Advanced Melanoma
Source: JAMA Netw Open. 2024 Aug 14;7(8):e2426641. doi: 10.1001/jamanetworkopen.2024.26641 (PMC11325208; doi:10.1001/jamanetworkopen.2024.26641)
Supplement: Supplement 1. — eFigure 1. Kaplan Meier Curve of Progression-Free Survival (PFS) of Patients With Advanced Melanoma Treated With Anti–PD-1, Stratified by Response, Calculated From the Date of Response eFigure 2. Kaplan Meier Curve of Overall Survival (OS) of Patients With Advanced Melanoma Treated With Anti–PD-1, Stratified by Response, Calculated From the Date of Response eFigure 3. Kaplan Meier Curve of Progression-Free Survival (PFS) of Patients With Advanced Melanoma Treated With Ipilimumab-Nivolumab, Stratified by Response, Calculated From the Date of Response eFigure 4. Kaplan Meier Curve of Overall Survival (OS) of Patients With Advanced Melanoma Treated With Ipilimumab-Nivolumab, Stratified by Response, Calculated From the Date of Response eFigure 5. Kaplan Meier Curve of Overall Survival (OS) of Patients With Advanced Melanoma Treated With Immune Checkpoint Inhibitors at Landmark Time From 3 Months After Start of Systemic Treatment, Stratified by Response Status at Landmark Time eFigure 6. Kaplan Meier Curve of Overall Survival (OS) of Patients With Advanced Melanoma Treated With Immune Checkpoint Inhibitors at Landmark Time From 6 Months After Start of Systemic Treatment, Stratified by Response Status at Landmark Time eTable 1. Best Objective Response Rate Stratified by Type of Systemic Treatment eTable 2. Patient and Tumor Characteristics of Patients With Advanced Melanoma Treated With Immune Checkpoint Inhibitors Stratified by Best Objective Response Rate: Complete or Partial Response vs No Complete or Partial Response eTable 3. Comparison of Findings of the Present Study With Other Relevant Trials eReferences. [file jamanetwopen-e2426641-s001.pdf]

Supplemental Online Content

van Not OJ, van den Eertwegh AJM, Jalving H, et al. Long-term survival in patients with advanced melanoma. *JAMA Netw Open*. 2024;e2426641.
doi:10.1001/jamanetworkopen.2024.26641

eFigure 1. Kaplan Meier Curve of Progression-Free Survival (PFS) of Patients With Advanced Melanoma Treated With Anti–PD-1, Stratified by Response, Calculated From the Date of Response

eFigure 2. Kaplan Meier Curve of Overall Survival (OS) of Patients With Advanced Melanoma Treated With Anti–PD-1, Stratified by Response, Calculated From the Date of Response

eFigure 3. Kaplan Meier Curve of Progression-Free Survival (PFS) of Patients With Advanced Melanoma Treated With Ipilimumab-Nivolumab, Stratified by Response, Calculated From the Date of Response

eFigure 4. Kaplan Meier Curve of Overall Survival (OS) of Patients With Advanced Melanoma Treated With Ipilimumab-Nivolumab, Stratified by Response, Calculated From the Date of Response

eFigure 5. Kaplan Meier Curve of Overall Survival (OS) of Patients With Advanced Melanoma Treated With Immune Checkpoint Inhibitors at Landmark Time From 3 Months After Start of Systemic Treatment, Stratified by Response Status at Landmark Time

eFigure 6. Kaplan Meier Curve of Overall Survival (OS) of Patients With Advanced Melanoma Treated With Immune Checkpoint Inhibitors at Landmark Time From 6 Months After Start of Systemic Treatment, Stratified by Response Status at Landmark Time

eTable 1. Best Objective Response Rate Stratified by Type of Systemic Treatment

eTable 2. Patient and Tumor Characteristics of Patients With Advanced Melanoma Treated With Immune Checkpoint Inhibitors Stratified by Best Objective Response Rate: Complete or Partial Response vs No Complete or Partial Response

eTable 3. Comparison of Findings of the Present Study With Other Relevant Trials

eReferences.

This supplemental material has been provided by the authors to give readers additional information about their work.

© 2024 van Not OJ et al. *JAMA Network Open.*

eFigure 1. Kaplan Meier Curve of Progression-Free Survival (PFS) of Patients With Advanced Melanoma Treated With Anti–PD-1, Stratified by Response, Calculated From the Date of Response

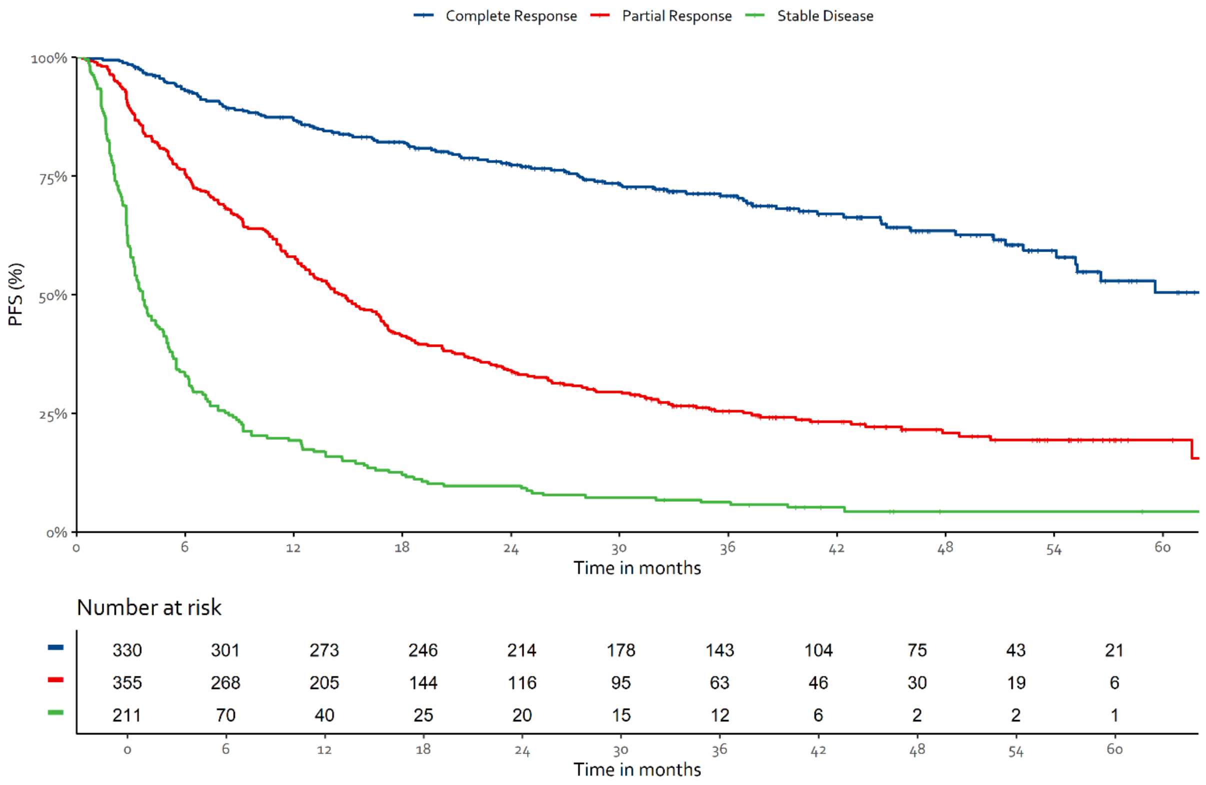

© 2024 van Not OJ et al. *JAMA Network Open.*

eFigure 2. Kaplan Meier Curve of Overall Survival (OS) of Patients With Advanced Melanoma Treated With Anti–PD-1, Stratified by Response, Calculated From the Date of Response

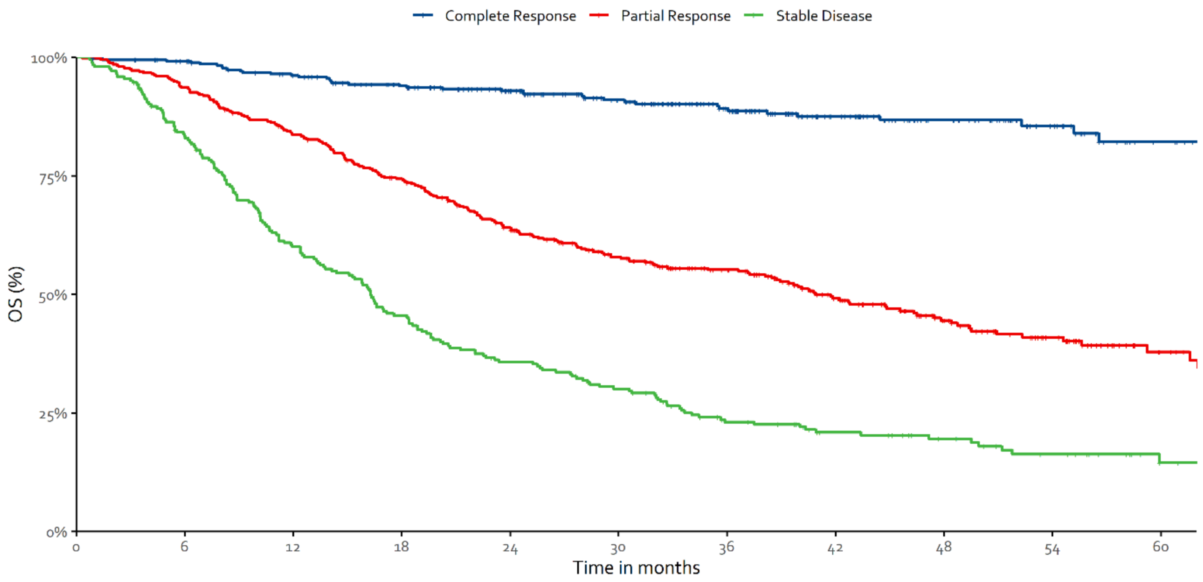

Number at risk

	0	6	12	18	24	30	36	42	48	54	60
	347	336	314	289	259	221	178	131	93	61	38
	392	367	325	284	239	200	162	119	83	55	25
	242	198	141	107	83	68	46	34	26	18	7

Time in months

© 2024 van Not OJ et al. *JAMA Network Open.*

eFigure 3. Kaplan Meier Curve of Progression-Free Survival (PFS) of Patients With Advanced Melanoma Treated With Ipilimumab-Nivolumab, Stratified by Response, Calculated From the Date of Response

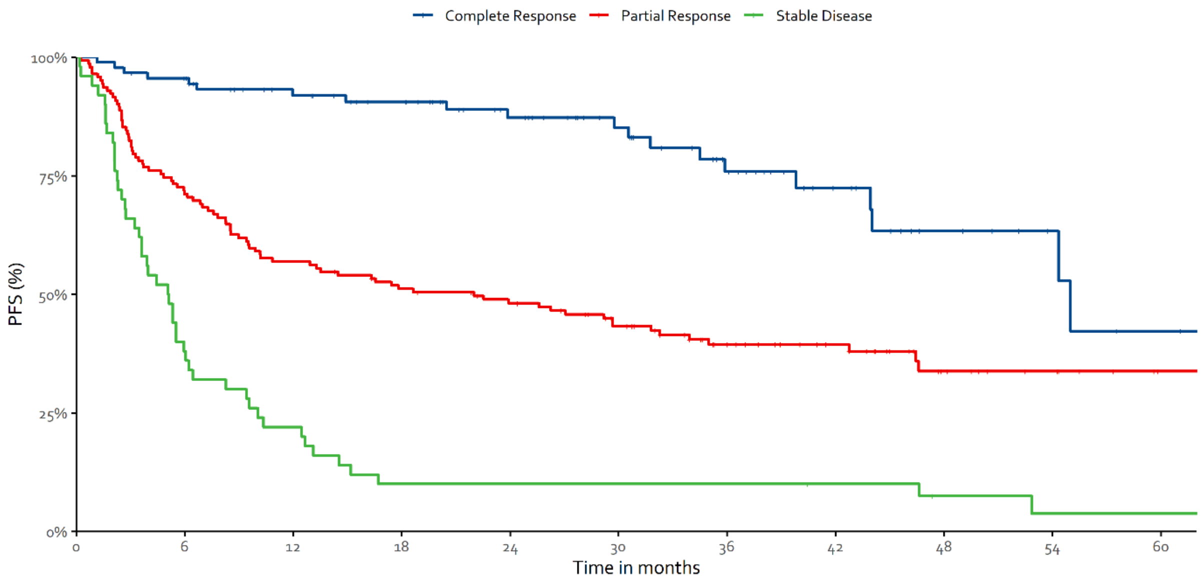

Number at risk

	0	6	12	18	24	30	36	42	48	54	60
	90	84	71	64	51	41	29	18	10	6	3
	142	101	80	70	63	51	36	27	14	8	2
	50	19	11	5	5	5	5	4	2	1	1

Time in months

© 2024 van Not OJ et al. *JAMA Network Open.*

eFigure 4. Kaplan Meier Curve of Overall Survival (OS) of Patients With Advanced Melanoma Treated With Ipilimumab-Nivolumab, Stratified by Response, Calculated From the Date of Response

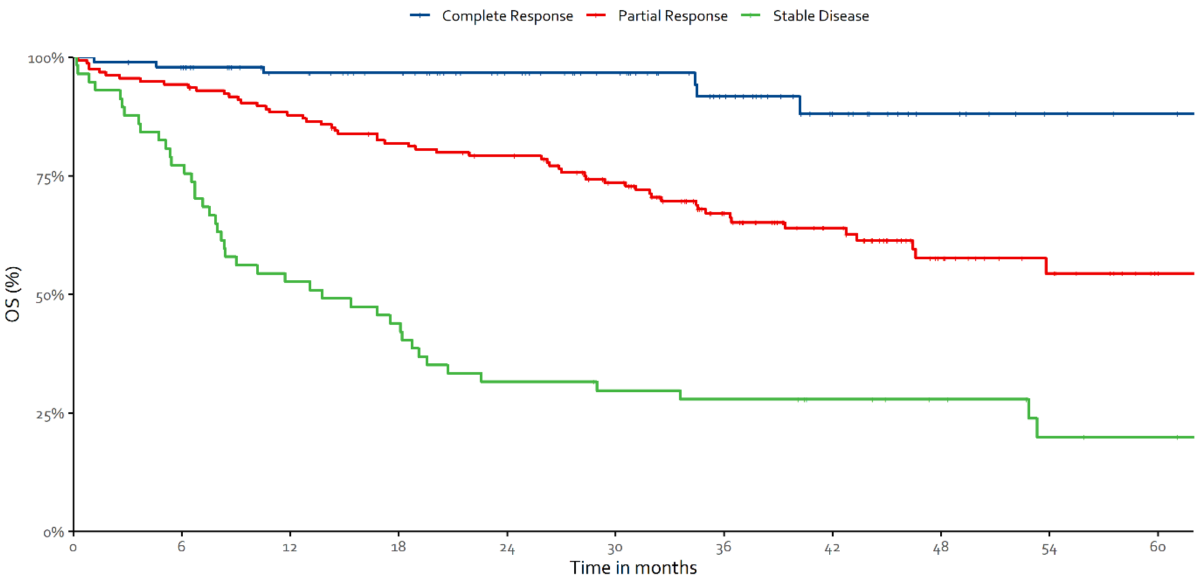

Number at risk

	0	6	12	18	24	30	36	42	48	54	60
(blue)	95	91	78	71	58	49	34	21	12	7	5
(red)	156	147	135	124	116	99	71	50	28	17	8
(green)	57	44	30	25	18	16	15	12	9	5	4

Time in months

© 2024 van Not OJ et al. *JAMA Network Open.*

eFigure 5. Kaplan Meier Curve of Overall Survival (OS) of Patients With Advanced Melanoma Treated With Immune Checkpoint Inhibitors at Landmark Time From 3 Months After Start of Systemic Treatment, Stratified by Response Status at Landmark Time

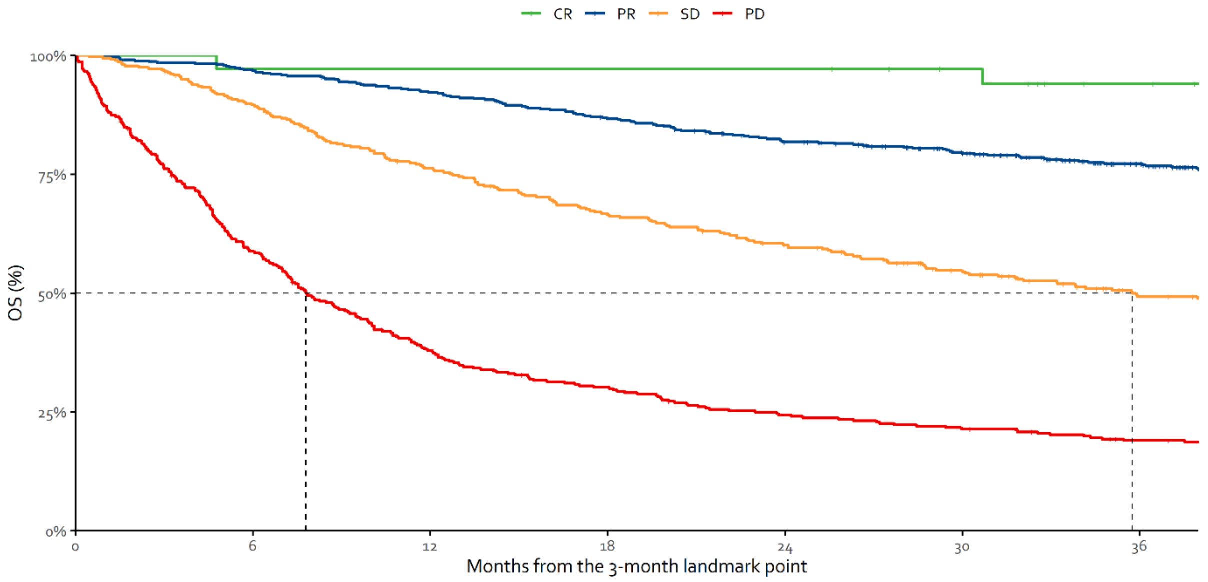

	0	6	12	18	24	30	36
CR	35	34	34	34	34	31	26
PR	525	508	483	452	419	384	317
SD	356	317	267	231	206	177	143
PD	357	207	132	104	83	73	60

Months from the 3-month landmark point

© 2024 van Not OJ et al. *JAMA Network Open.*

eFigure 6. Kaplan Meier Curve of Overall Survival (OS) of Patients With Advanced Melanoma Treated With Immune Checkpoint Inhibitors at Landmark Time From 6 Months After Start of Systemic Treatment, Stratified by Response Status at Landmark Time

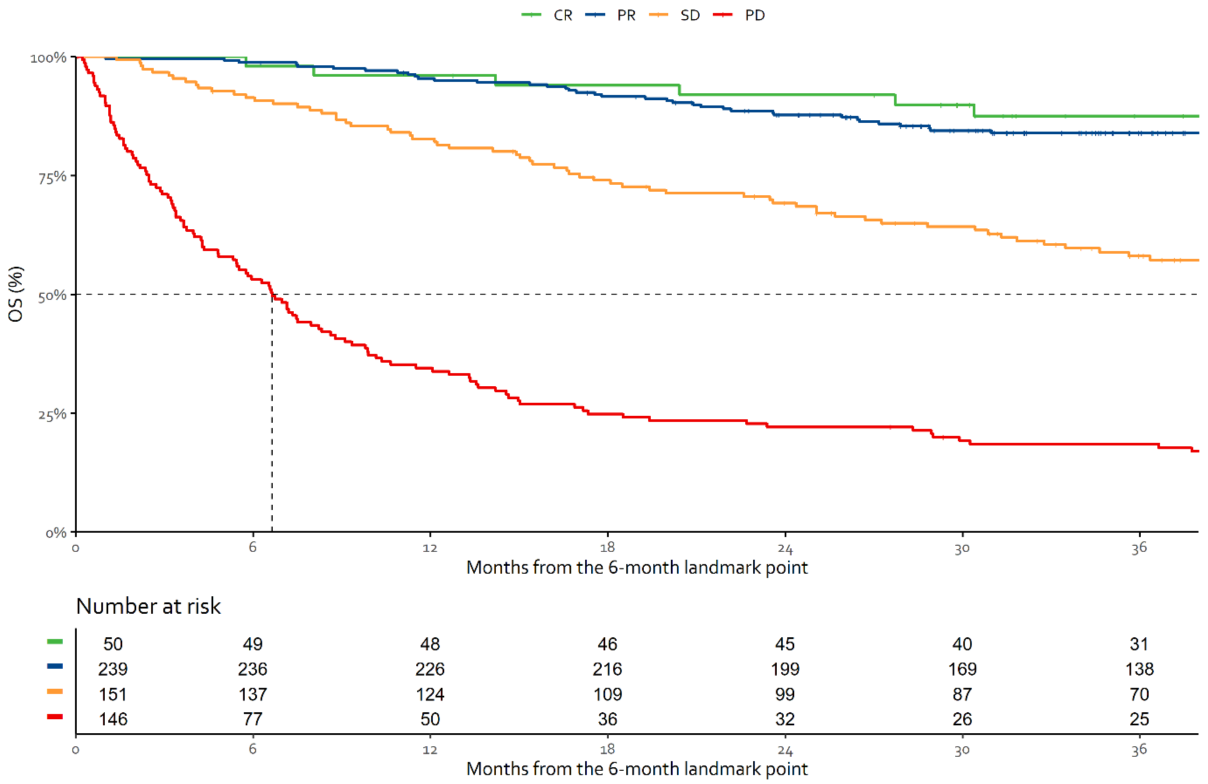

© 2024 van Not OJ et al. *JAMA Network Open.*

eTable 1. Best Objective Response Rate Stratified by Type of Systemic Treatment

Best objective response rate	Ipilimumab	Anti–PD-1	Ipilimumab-nivolumab	Total
**Complete response**	47 (7.8%)	357 (25.3%)	100 (20.8%)	504 (20.2%)
**Partial response**	58 (9.7%)	392 (27.8%)	156 (32.5%)	606 (24.3%)
**Stable disease**	208 (34.6%)	242 (17.2%)	57 (11.9%)	507 (20.4%)
**Progressive disease**	253 (42.1%)	342 (24.3%)	123 (25.6%)	718 (28.8%)
**Death**	29 (4.8%)	70 (5.0%)	38 (7.9%)	137 (5.5%)
**NA**	6 (1.0%)	6 (0.4%)	6 (1.2%)	18 (0.7%)

© 2024 van Not OJ et al. *JAMA Network Open.*

eTable 2. Patient and Tumor Characteristics of Patients With Advanced Melanoma Treated With Immune Checkpoint Inhibitors Stratified by Best Objective Response Rate: Complete or Partial Response vs No Complete or Partial Response

		Complete or partial response	No complete or partial response	P-value
**N**		1110	1380	
**Age (%)**	<65 years	523 (47.1)	657 (47.6)	0.839
	≥65 years	587 (52.9)	723 (52.4)	
**Median age [IQR]**		66.0 [55.0, 73.0]	65.0 [56.0, 73.0]	0.550
**Sex (%)**	Male	684 (61.6)	877 (63.6)	0.343
	Female	426 (38.4)	503 (36.4)	
**ECOG PS (%)**	0	670 (60.4)	731 (53.0)	<0.001
	1	331 (29.8)	470 (34.1)	
	≥2	43 (3.9)	92 (6.7)	
	Unknown	66 (5.9)	87 (6.3)	
**LDH levels (%)**	Not determined	15 (1.4)	18 (1.3)	<0.001
	Normal	815 (73.4)	900 (65.2)	
	1-2x ULN	225 (20.3)	327 (23.7)	
	>2x ULN	51 (4.6)	129 (9.3)	
**Liver metastases (%)**	No	879 (79.2)	960 (69.6)	<0.001
	Yes	222 (20.0)	404 (29.3)	
	Unknown	9 (0.8)	16 (1.2)	
**Brain metastases (%)**	No	874 (78.7)	1043 (75.6)	0.002
	Yes, asymptomatic	156 (14.1)	175 (12.7)	
	Yes, symptomatic	78 (7.0)	156 (11.3)	
	Unknown	2 (0.2)	6 (0.4)	
**AJCC stage (8th edition) (%)**	III irresectable	86 (7.7)	69 (5.0)	<0.001
	IV-M1a	133 (12.0)	124 (9.0)	
	IV-M1b	193 (17.4)	183 (13.3)	
	IV-M1c	462 (41.6)	667 (48.3)	
	IV-M1d	234 (21.1)	331 (24.0)	

© 2024 van Not OJ et al. *JAMA Network Open.*

	Unknown	2 (0.2)	6 (0.4)	
**Organ sites (%)**	<3	667 (60.1)	724 (52.5)	0.001
	≥3	438 (39.5)	650 (47.1)	
	Unknown	5 (0.5)	6 (0.4)	

© 2024 van Not OJ et al. *JAMA Network Open.*

eTable 3. Comparison of Findings of the Present Study With Other Relevant Trials

Progression-Free Survival	3 years	5 years	Percentage experiencing progression, death or event between year 3 and 5
**Ipilimumab**			
**DMTR**	8%	7%	1%
**CheckMate 067[1,2]**	10%	8%	2%
**Anti–PD-1**			
**DMTR**	29%	24%	5%
**CheckMate 067[1,2]**	32%	29%	3%
**KEYNOTE 001[3]**		29%	
**Ipilimumab-nivolumab**			
**DMTR**	34%	28%	6%
**CheckMate 067[1,2]**	39%	36%	3%
**Overall Survival**	**3 years**	**5 years**	**Percentage experiencing death or event between year 3 and 5**
**Ipilimumab**			
**DMTR**	32%	25%	7%
**CheckMate 067[1,2]**	34%	26%	8%
**Anti–PD-1**			
**DMTR**	47%	39%	8%
**CheckMate 067[1,2]**	52%	44%s	8%
**KEYNOTE 001[3]**	45%	41%	4%
**Ipilimumab-nivolumab**			
**DMTR**	50%	41%	9%
**CheckMate 067[1,2]**	58%	52%	6%

© 2024 van Not OJ et al. *JAMA Network Open.*

eReferences

1. Wolchok JD, Chiarion-Sileni V, Gonzalez R, et al. Overall Survival with Combined Nivolumab and Ipilimumab in Advanced Melanoma. N Engl J Med. 2017;377(14):1345-1356. doi:10.1056/nejmoa1709684

2. Larkin J, Chiarion-Sileni V, Gonzalez R, et al. Five-Year Survival with Combined Nivolumab and Ipilimumab in Advanced Melanoma. N Engl J Med. 2019;381(16):1535-1546. doi:10.1056/nejmoa1910836

3. Hamid O, Robert C, Daud A, et al. Five-year survival outcomes for patients with advanced melanoma treated with pembrolizumab in KEYNOTE-001. Ann Oncol. 2019;30(4):582-588. doi:10.1093/annonc/mdz011

© 2024 van Not OJ et al. *JAMA Network Open.*
