# Supplementary material for: Long-Term Survival in Patients With Advanced Melanoma
Source: JAMA Netw Open. 2024 Aug 14;7(8):e2426641. doi: 10.1001/jamanetworkopen.2024.26641 (PMC11325208; doi:10.1001/jamanetworkopen.2024.26641)
Supplement: Supplement 2. — Data Sharing Statement [file jamanetwopen-e2426641-s002.pdf]

Data Sharing Statement

van Not. Long-Term Survival In Patients With Advanced Melanoma. *JAMA Netw Open*. Published August 14, 2024. doi:10.1001/jamanetworkopen.2024.26641

Data
Data available: No

Additional Information
Explanation for why data not available: Data can be made available upon reasonable request to the corresponding author.
